# Impact of Biannual Azithromycin on Anemia in Preschool Children in Kilosa District, Tanzania: A Cluster-Randomized Clinical Trial

**DOI:** 10.4269/ajtmh.19-0500

**Published:** 2020-02-17

**Authors:** Evan M. Bloch, Beatriz Munoz, Jerusha Weaver, Zakayo Mrango, Thomas M. Lietman, Sheila K. West

**Affiliations:** 1 Department of Pathology, Johns Hopkins School of Medicine, Baltimore, Maryland;; 2 Dana Center for Preventive Ophthalmology, Johns Hopkins School of Medicine, Baltimore, Maryland;; 3 National Institute for Medical Research, Kilosa, Tanzania;; 4 Francis I Proctor Foundation, University of California, San Francisco, San Francisco, California

## Abstract

A cluster-randomized clinical trial showed that biannual single-dose azithromycin reduced mortality in preschool children; we sought to determine the effect on anemia. A simple random sample of 30 communities from Kilosa district, Tanzania, were themselves randomized to receive either 6-monthly treatment of children aged 1–59 months with single-dose azithromycin or placebo. From each community, 40 preschool children were randomly selected at baseline, 12 months, and 24 months. At surveys, the children underwent hemoglobin testing; WHO definitions for anemia were applied. After adjusting for community clustering, the prevalence of anemia was not significantly different by treatment assignment at baseline, 12 months, and 24 months. In each of the cross-sectional surveys, anemia prevalence was associated with younger age; the odds of being anemic was highest in those aged < 12 months. There was also a general decrease in the prevalence of anemia during the study. Although azithromycin was not shown to affect anemia, significantly, the study highlights burden of anemia in rural, African communities.

Anemia refers to a diverse group of disorders characterized by the shared inability to meet oxygen demand on the basis of insufficient red cell mass or impaired function.^1^ Rather than a single disease, anemia represents a clinical sign, a manifestation of multiple different pathologies.^2^ As such, anemia is classified in a variety of different ways spanning mechanism (i.e., production, destruction/consumption, and blood loss/bleeding), morphology (i.e., micro-, normo- versus macrocytic), and iron status (i.e., hypo- versus normochromic). Anemia remains a major global health challenge, with more than 1.5 billion people affected.^3^ Preschool children (i.e., those aged 6 months–5 years) in low-middle income countries are disproportionately at risk of anemia,^3^ as illustrated by a 2008 report that 47% of preschool children worldwide were anemic.^3^ Anemia in childhood has long been postulated to adversely affect behavioral, cognitive, and psychomotor development^4–6^ as well as mortality risk. Therefore, interventions that tackle anemia could offer protection against childhood morbidity.^7^

The Macrolides Oraux pour Réduire les Décès avec un Oeil sur la Résistance (MORDOR) study, a multinational cluster randomized clinical trial, found that biannual administration of single-dose azithromycin to preschool children reduced all-cause mortality compared with placebo.^8^ Mechanisms for decreased mortality with azithromycin were explored but not identified.^9^ Specifically, selected morbidity indicators (e.g., cough, fever, and diarrhea) were not significantly different by treatment assignment in a cohort of children in the participating communities.^9^ Azithromycin is a broad-spectrum macrolide antibiotic that has proven to be safe and effective against a wide array of respiratory-,^10^ gastrointestinal-,^11^ and cutaneous pathogens,^11^ many of which are encountered in low-resourced settings. It is also effective against selected protozoan infections such as *Babesia.*^12^ Importantly, mass distribution of single-dose azithromycin has been central to the global strategy to eliminate trachoma^13^ and yaws.^14^ Acknowledging that there is a complex interplay between anemia and infectious disease,^15^ we hypothesized that azithromycin could—indirectly—impact the prevalence of anemia through mitigation of infectious burden in a rural, population of children of low socioeconomic status. We sought to determine, in a parallel randomized trial, whether biannual distribution of azithromycin to preschool children impacted the prevalence of anemia compared with placebo in Kilosa district, Tanzania.

A cluster-randomized, placebo-controlled, double-masked clinical trial was conducted in 30 communities in Kilosa District, Tanzania (January 2015–August 2017), as part of the MORDOR trial, to evaluate the effect of biannual, single-dose azithromycin compared with placebo on the prevalence of anemia over time. All communities that were located in Kilosa district that had at least 20 children aged 1–59 months during a baseline census were eligible to participate in the trial. Thirty communities were randomly selected^8^ from the same pool as that of the MORDOR mortality study (i.e., enabling inference to the MORDOR population) but were not included in that study. The intervention, azithromycin (∼20 mg/kg), and placebo (Pfizer, New York, NY) (both of which were indistinguishable in appearance and taste) were administered every 6 months for 24 months, by study personnel. At each survey time point, an updated census was used: 40 children, aged 1–59 months who had guardians who were capable of providing consent, were randomly selected to participate in the study. If a community had less than 40 eligible children, all the children were selected to participate. A fingerstick was performed on each of the children and the hemoglobin concentration was determined using a point-of-care device HemoCue^®^ Hb 201+ System (HemoCue America, Brea, CA).

The prespecified outcome of interest was the prevalence of mild (10–10.9 g/dL), moderate (7–9.9 g/dL), and severe anemia (< 7 g/dL) as defined by the WHO classification of anemia.^1^ Descriptive statistics for all outcomes stratified by treatment arm are shown for the cross-sectional surveys; 95% CIs are shown for the primary outcome (anemia). Cross-sectional differences and changes in prevalence over time by arm were tested using logistic models with arm age and time of survey as independent predictors as appropriate. The generalized estimating equation approach, with an independent correlation structure, was used to account for clustering at the community level. Data were analyzed with SAS version 9.4 software (SAS, Raleigh, NC).

Ethical approval was obtained from the Tanzanian National Institute for Medical Research and the Institutional Review Board of the Johns Hopkins School of Medicine. Children were included in the study on the basis of documented written informed consent from their guardians.

After adjusting for age and accounting for clustering at a community level, the prevalence of anemia as assessed at baseline, 12 months, and 24 months was not significantly different between children who resided in communities in the azithromycin and the placebo arm (Table 1). The proportion of children with anemia was also not significantly different by treatment assignment after adjusting for the number of doses of the study drug (Table 2). In both treatment arms (i.e., azithromycin and placebo), there was an overall decline in the prevalence of anemia over the course of the study as shown in the 12-month and 24-month follow-up surveys (Table 1) (odds ratio [OR] = 0.56; 95% CI [0.48–0.66] per year). The prevalence of anemia also declined with age; it was significantly higher in those children aged < 12 months and remained significantly higher in the youngest age stratum (as compared with other ages) through all phases of follow-up: at baseline (OR = 2.71; 95% CI [1.84–4.00]), at 12 months (OR = 2.15; 95% CI [1.50–3.06]), and at 24 months (OR = 1.70; 95% CI [1.01–2.88]). In most cases, anemia was mild or moderate; by comparison, severe anemia was rare (0.2–1%) in all phases of follow-up (Figure 1). The median hemoglobin value (and interquartile ranges) at baseline, 12 months, and 24 months in the azithromycin arm was 10.65 g/dL (9.80–11.50), 11.00 g/dL (10.10–11.80), and 11.40 g/dL (10.60–12.20), respectively. The median hemoglobin value (and interquartile ranges) at baseline, 12 months, and 24 months in the placebo arm were 10.60 g/dL (9.60–11.40), 10.90 g/dL (10.00–11.70), and 11.30 g/dL(10.50–12.20), respectively.

**Table 1:** Proportion of children with moderate to severe anemia by survey time age and arm

Survey time	Age category	Arm		Arm differences; age-adjusted odds ratio 95% CI; *P*-value; reference placebo*
		Azithromycin (*n*/*N*) (%)	Placebo (*n*/*N*) (%)	
Baseline	1 to < 12 months	44/94 (46.8)	45/86 (52.3)	0.78 (0.46–1.33), 0.36
	12 to < 24 months	50/118 (42.4)	50/105 (47.6)	
	24 to < 36 months	16/92 (17.4)	28/112 (25.0)	
	36 to < 48 months	21/102 (20.6)	25/107 (23.4)	
	48 to 59 months	13/98 (13.3)	24/115 (20.9)	
	Overall	144/504 (28.6)	172/525 (32.8)	
	Test for trend with age adjusted for arm*	< 0.0001		
12 months	< 12 months	43/130 (33.1)	33/96 (34.4)	0.81 (0.53–1.26), 0.36
	12 to < 24 months	32/143 (22.4)	32/130 (24.6)	
	24 to < 36 months	26/135 (19.3)	34/140 (24.3)	
	36 to < 48 months	12/101 (11.9)	23/117 (19.7)	
	48 to 59 months	9/80 (11.3)	8/63 (12.7)	
	Overall	122/589 (20.7)	130/546 (23.8)	
	Test for trend with age adjusted for arm*	< 0.0001		
24 months	< 12 months	10/75 (13.3)	14/60 (23.3)	0.74 (0.40–1.37),0.33
	12 to < 24 months	19/132 (14.4)	25/113 (22.1)	
	24 to < 36 months	12/105 (11.3)	15/125 (12.0)	
	36 to < 48 months	3/63 (4.8)	6/87 (6.9)	
	48 to 59 months	6/70 (8.6)	5/98 (5.1)	
	Overall	50/445 (11.2)	65/483 (13.5)	
	Test for trend with age adjusted for arm*	0.001		

* Accounting for clustering at community level.

**Table 2:** Proportion of children with moderate-to-severe anemia by survey time and number of previous treatments with the study drug

Survey time	Number of previous treatments with the study drug	Arm		Age-adjusted treatment-adjusted *P*-value for arm differences*
		Azithromycin (*n*/*N*) (%)	Placebo (*n*/*N*) (%)	
Baseline	0	144/504 (28.6)	172/525 (32.8)	0.36
12 months	0	26/102 (25.5)	28/95 (29.5)	0.36
	1	42/160 (26.3)	40/146 (27.4)	
	2	54/327 (16.5)	62/305 (20.3)	
	Age-adjusted *P*-value for number of treatments*	0.06	0.38	
24 months	0	5/32 (15.6)	5/44 (11.4)	0.38
	1	11/49 (22.5)	14/54 (25.9)	
	2	6/79 (7.6)	18/88 (18.2)	
	3	15/103 (14.6)	16/134 (11.9)	
	4	13/182 (7.1)	14/163 (8.6)	
	Age-adjusted *P*-value for number of treatments*	0.12	0.33	

* Accounting for clustering at community level.

**Figure 1.**
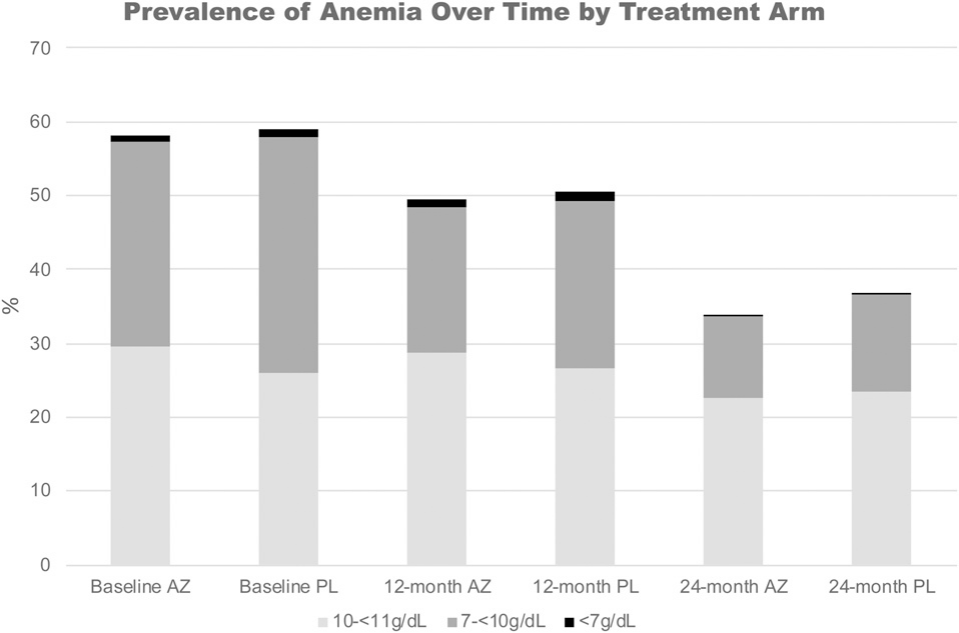
Prevalence of anemia by treatment assignment at baseline, 12 months, and 24 months.

The study findings were 3-fold. First, biannual administration of azithromycin to preschool children did not have a significant impact on prevalence of anemia over time. Acknowledging differences in study design, similar negative findings were reported in cluster-randomized trials in Niger when evaluating the impact of mass distribution of azithromycin on malaria parasitemia.^16–18^ Second, there was a significant decline in anemia by age, which was shown in both treatment groups. The prevalence of anemia was significantly higher in those aged less than 1 year. The reasons for this finding are not entirely clear as breastfeeding is highly prevalent in young children. Early weaning from breastfeeding with transition to low-nutrition foods may be one possible—albeit uninvestigated—explanation for higher rates of anemia in the youngest age stratum, if confirmed, this could be amenable to intervention (breastfeeding data were not collected in our study). A similar finding was reported in a study of preschool children in rural Rwanda, where children aged 6–11 months had the highest odds of moderate to severe anemia.^19^ By contrast, malaria was associated with higher age-groups. Third, although a significant difference by intervention was not shown, the study still highlights the high prevalence of anemia in a rural district of Tanzania, whereby almost a third of all children were moderately to severely anemic at baseline. Regional studies have shown even higher rates of anemia. One study in Arusha district reported that 84.6% of a random sample of 369 children aged < 5 years were anemic.^20^ Common, regional contributing risk factors for anemia in children include low birth weights, dietary factors (e.g., inadequate bioavailability of dietary iron), malaria, schistosomiasis, and soil-transmitted helminths.^20,21^ Iron deficiency specifically contributes disproportionately to the burden of anemia, being present in 42% of all cases of anemia in children.^21^

Nonetheless, there was a decline in the prevalence of anemia in both arms over the course of the study. Such is consistent with findings that have been reported at a national level over the past 15 years.^22^ For example, in 2004–2005, the national prevalence rates of moderate and severe anemia in Tanzanian children aged 6 months to 5 years were 43% and 4.2%, respectively; by 2010, those prevalence rates had declined to 29.4% and 1.9%, respectively.^22^ Similar downward trends in the prevalence of anemia have been reported for the broader East Africa region, where the regional prevalence of severe anemia decreased from 10.2% in 1995 to 2.5% in 2011.^21^ One plausible explanation for some of the decline is a contemporaneous reduction in malaria during the study, following concerted efforts to mitigate malaria through distribution of insecticide-treated bed nets and expanded access to antimalarials.^23^

The study has limitations. For one, anemia is not a diagnosis. Rather it is a clinical sign with multifactorial origin. Pertinent to this study, assessment of anemia was limited to only one parameter. Although this offers insight into prevalence, it does little to characterize the cause of anemia. Separate from the study’s goal to evaluate the impact of azithromycin on anemia, improved understanding of the nature of anemia is necessary to understand the broader changes in Tanzania, so as to guide intervention accordingly.

In conclusion, anemia remains a major public health burden in preschool children in Kilosa district, Tanzania. However, biannual single-dose azithromycin treatment was not shown to impact the prevalence of anemia. The lack of significant effect on anemia in azithromycin-treated communities is consistent with negative mortality finding as previously reported for Tanzania.^8^

## References

[1] WHO, 2011 Haemoglobin concentrations for the diagnosis of anaemia and assessment of severity. Vitamin and Mineral Nutrition Information System. Geneva, Switzerland: World Health Organization.

[2] Cappellini MD Motta I, 2015 Anemia in clinical practice-definition and classification: does hemoglobin change with aging? Semin Hematol 52: 261–269. 26404438 10.1053/j.seminhematol.2015.07.006

[3] McLean E Cogswell M Egli I Wojdyla D de Benoist B, 2009 Worldwide prevalence of anaemia, WHO vitamin and mineral nutrition information system, 1993–2005. Public Health Nutr 12: 444–454. 18498676 10.1017/S1368980008002401

[4] Lozoff B De Andraca I Castillo M Smith JB Walter T Pino P, 2003 Behavioral and developmental effects of preventing iron-deficiency anemia in healthy full-term infants. Pediatrics 112: 846–854. 14523176

[5] Stoltzfus RJ, 2001 Iron-deficiency anemia: reexamining the nature and magnitude of the public health problem. Summary: implications for research and programs. J Nutr 131: 697S–700S; discussion 700S–701S. 11160600 10.1093/jn/131.2.697S

[6] Congdon EL Westerlund A Algarin CR Peirano PD Gregas M Lozoff B Nelson CA, 2012 Iron deficiency in infancy is associated with altered neural correlates of recognition memory at 10 years. J Pediatr 160: 1027–1033. 22244466 10.1016/j.jpeds.2011.12.011 PMC3360801

[7] Vaivada T Gaffey MF Bhutta ZA, 2017 Promoting early child development with interventions in health and nutrition: a systematic review. Pediatrics 140: e20164308. 28771408 10.1542/peds.2016-4308

[8] Keenan JD 2018 Azithromycin to reduce childhood mortality in sub-Saharan Africa. N Engl J Med 378: 1583–1592. 29694816 10.1056/NEJMoa1715474 PMC5849140

[9] West SK Bloch E Weaver J Munoz B Mrango Z Kasubi M Lietman T Coles C, 2019 Morbidity in a longitudinal cohort of children residing in villages randomized to 6 monthly treatment with azithromycin versus placebo. Clin Infect Dis ciz269. 10.1093/cid/ciz269 30950493

[10] Coles CL Levens J Seidman JC Mkocha H Munoz B West S, 2012 Mass distribution of azithromycin for trachoma control is associated with short-term reduction in risk of acute lower respiratory infection in young children. Pediatr Infect Dis J 31: 341–346. 22173140 10.1097/INF.0b013e31824155c9

[11] Fry AM Jha HC Lietman TM Chaudhary JS Bhatta RC Elliott J Hyde T Schuchat A Gaynor B Dowell SF, 2002 Adverse and beneficial secondary effects of mass treatment with azithromycin to eliminate blindness due to trachoma in Nepal. Clin Infect Dis 35: 395–402. 12145722 10.1086/341414

[12] Krause PJ 2000 Atovaquone and azithromycin for the treatment of babesiosis. N Engl J Med 343: 1454–1458. 11078770 10.1056/NEJM200011163432004

[13] Melese M 2004 Feasibility of eliminating ocular *Chlamydia trachomatis* with repeat mass antibiotic treatments. JAMA 292: 721–725. 15304470 10.1001/jama.292.6.721

[14] Mitja O 2015 Mass treatment with single-dose azithromycin for yaws. N Engl J Med 372: 703–710. 25693010 10.1056/NEJMoa1408586

[15] Drakesmith H Prentice AM, 2012 Hepcidin and the iron-infection axis. Science 338: 768–772. 23139325 10.1126/science.1224577

[16] Gaynor BD 2014 Impact of mass azithromycin distribution on malaria parasitemia during the low-transmission season in Niger: a cluster-randomized trial. Am J Trop Med Hyg 90: 846–851. 24615132 10.4269/ajtmh.13-0379 PMC4015576

[17] O'Brien KS 2017 Mass azithromycin and malaria parasitemia in Niger: results from a community-randomized trial. Am J Trop Med Hyg 97: 696–701. 28722569 10.4269/ajtmh.16-0487 PMC5590561

[18] Oldenburg CE 2018 Annual versus biannual mass azithromycin distribution and malaria parasitemia during the peak transmission season among children in Niger. Pediatr Infect Dis J 37: 506–510. 29088030 10.1097/INF.0000000000001813 PMC5924654

[19] Kateera F Ingabire CM Hakizimana E Kalinda P Mens PF Grobusch MP Mutesa L van Vugt M, 2015 Malaria, anaemia and under-nutrition: three frequently co-existing conditions among preschool children in rural Rwanda. Malar J 14: 440. 26542672 10.1186/s12936-015-0973-z PMC4635556

[20] Kejo D Petrucka PM Martin H Kimanya ME Mosha TC, 2018 Prevalence and predictors of anemia among children under 5 years of age in Arusha District, Tanzania. Pediatr Health Med Ther 9: 9–15. 10.2147/PHMT.S148515 PMC5804135 29443328

[21] Stevens GA Finucane MM De-Regil LM Paciorek CJ Flaxman SR Branca F Pena-Rosas JP Bhutta ZA Ezzati M; Nutrition Impact Model Study G, 2013 Global, regional, and national trends in haemoglobin concentration and prevalence of total and severe anaemia in children and pregnant and non-pregnant women for 1995–2011: a systematic analysis of population-representative data. Lancet Glob Health 1: e16–e25. 25103581 10.1016/S2214-109X(13)70001-9 PMC4547326

[22] USAID TANZANIA National Anemia Profile.

[23] NBS, 2017 Tanzania Malaria Indicator Survey Key Indicators. Dar es Salaam, Tanzania: The National Bureau of Statistics (NBS).

